# To adjust or not to adjust: Cut-off scores in performance validity testing in Portuguese older adults with dementia

**DOI:** 10.3389/fpsyg.2022.989432

**Published:** 2022-08-11

**Authors:** Sandra Fernandes, Inês Ferreira, Luís Querido, Julia C. Daugherty

**Affiliations:** ^1^Faculdade de Psicologia, Universidade de Lisboa, Lisbon, Portugal; ^2^CICPSI, Faculdade de Psicologia, Universidade de Lisboa, Lisbon, Portugal; ^3^Centro de Investigação Interdisciplinar Egas Moniz (CiiEM), Instituto Universitário Egas Moniz (IUEM), Caparica, Portugal; ^4^Psychology Department, Campus Duques de Soria, University of Valladolid, Valladolid, Spain

**Keywords:** performance validity tests, simulation, cut-offs, older adults, dementia

## Abstract

The rising demographic of older adults worldwide has led to an increase in dementia cases. In order to ensure the proper allocation of care and resources to this clinical group, it is necessary to correctly distinguish between simulated versus bona-fide cognitive deficits typical of dementia. Performance Validity Tests (PVTs) are specifically designed to assess a lack of effort and the possible simulation of cognitive impairment. Previous research demonstrates that PVTs may be sensitive to dementia, thus inaccurately classifying real memory impairment as simulation. Here, we analyzed the sensitivity of PVTs in discriminating between dementia and simulation using receiver operating characteristic (ROC) curve analyses. Further, we examined the potential need for adjusting cut-off scores for three stand-alone (Test of Memory Malingering, Rey-15 Item Memory Test, and Coin in Hand-Extended Version) and one embedded (Reliable Digit Span) PVT for Portuguese older adults with dementia. The results showed that (1) all measures, except for the Coin in Hand— Extended version (CIH-EV), were sensitive to one or more sociodemographic and/or cognitive variables, and (2) it was necessary to adjust cut-off points for all measures. Additionally, the Rey-15 Item Memory Test did not demonstrate sufficient discriminating capacity for dementia. These results present important implications for clinical practice and the daily life of patients, as the use of incorrect cut-off points could impede patients from getting the resources they need.

## Introduction

We are currently witnessing the progressive aging of the world population. By 2050, it is estimated that 1 in every six individuals will be older than 65, a stark increase from the 2019 estimate of 1 in 11 (United Nations, 2019). Several critical implications have emerged from this “longevity revolution.” Among them, there is a growing concern for adjusting national social policies related to retirement age, as has happened in Portugal in recent years (Decreto-Lei n.º 119/2018 de 27 de Dezembro do Ministério do Trabalho, Solidariedade e Segurança Social, 2018). An additional concern is the expected increase in neurodegenerative diseases, considering age is the biggest risk factor for dementia (Alzheimer’s Society, 2016).

Dementia is a progressive pathology associated with aging in which one or more cognitive functions decline from a previous level of functioning beyond what is expected for their age and education (American Psychiatric Association, 2013), compromising autonomy in Activities of Daily Living. In response to the increase in dementia prevalence and difficulties in working beyond retirement age, early retirement requests have risen. In fact, in Portugal, 18,725 people benefited from early retirement in 2010, a number that rose to over 150,000 in 2016 and in 2019 (161,530 and 152,369, respectively; Instituto Nacional de Estatística [INE], 2021).

In addition, with the demographic rise of older populations and the progressive increment of older adult dependency, institutionalization in Residential Care Structures is increasing (Toth et al., 2022). Due to the long wait times and difficulties in accessing these specialized structures, individuals may simulate cognitive deficits or claim incapacity to obtain a diagnosis that facilitates entry. Other reasons for simulating cognitive deficits akin to dementia include acquiring attributions and/or a higher value for early retirement subsidies. Dementia simulation has many consequences, draining limited Social Security resources and inappropriately depriving resources from those who really need them (Yeh et al., 2019). For these reasons, there is an urgent need to distinguish between real and simulated cognitive deficits.

The distinction can be made using either embedded or stand-alone Performance Validity Tests (PVTs), which detect inconsistent or invalid response patterns. Embedded PVTs, as their name suggests, are “built-into” existing neuropsychological tests (e.g., Reliable Digit Span from the Wechsler Adult Intelligence Scale) on which e-values tend to demonstrate a psychometric floor performance. As such, scores below the floor level are more likely to reflect a lack of effort or simulation. Stand-alone PVTs, on the other hand, do not form a part of traditional cognitive tests and are specifically designed to assess invalid/valid responses and/or simulation on their own (Greher and Wodushek, 2017). An additional common characteristic of many PVTs is the forced-choice response paradigm, in which participants are “forced” to choose between two responses (e.g., Test of Memory Malingering, the Coin in Hand-Extended Version, and Rey-15 Item Memory Test; Rudman et al., 2011).

To improve incremental predictive validity and performance credibility (Nelson et al., 2003), the current recommendation is to use multiple PVTs in conjunction with one another (Heilbronner et al., 2009). However, in Portugal, and in many countries across the globe, this is difficult because there are a limited number of validated PVTs for older adults [e.g., only three in Portugal: the Test of Memory Malingering (TOMM), the Rey 15-item Memory Test (REY-15IMT), and the Coin in Hand— Extended version (CIH-EV)]. Further, although the TOMM and the REY-15IMT are adequate for Mild Cognitive Decline (MCI; e.g., Fernandes, 2009; Pinho, 2012); evidence suggests they may not be for dementia (e.g., Dean et al., 2009; Ferreira et al., 2022). Research has shown that the diagnostic accuracy of PVTs decreases as the severity of dementia increases (McGuire et al., 2019), and that the TOMM and the Rey-15IMT may be the most sensitive measures for cognitive deterioration in dementia patients (e.g., Rudman et al., 2011). Due to these difficulties, dementia groups are often excluded from validation or replication studies with PVTs (Dean et al., 2009). Thus, results on this population are rare and often inconsistent (Rudman et al., 2011). This is a worrying scenario, given the increasing aging population, prevalence of dementia, requests for early retirement (Instituto Nacional de Estatística [INE], 2021), and waiting lists to access residential structures for older people.

Some suggest that the most practical solution would be to adjust the cut-off points (Van Gorp and Hassenstab, 2009; Simões et al., 2010). Following the recommended guidelines outlined by Sugarman and Axelrod (2015), a commonly used criterion is to achieve at least 50% sensitivity and 90% specificity for the selected cut-off points. Thus, the present study aims to (i) compare the sensitivity of PVTs validated in Portugal [Reliable Digit Span (RDS), TOMM, REY-15IMT, and CIH-EV] to demographic variables and cognitive functioning; (ii) assess the need for and adequacy of cut-off adjustments for these instruments for dementia patients in Portugal; and (iii) select the best cut-offs for a clinical sample of dementia patients using guidelines provided by Sugarman and Axelrod (2015).

Considering previous studies have pointed to the influence of sociodemographic and neurocognitive variables on the TOMM (e. g., Pinho, 2012), REY-15IMT (e. g., Silva, 2018), and RDS (Zenisek et al., 2016), but not on the CIH-EV (Ferreira et al., 2022), it is expected that all measures, except for the CIH-EV, will be sensitive to at least one sociodemographic variable and cognitive deterioration (H1). Previous research also indicates the need to adjust cut-off points for groups with dementia (e.g., McGuire et al., 2019). As such, we expect adjustments will be necessary and appropriate for all measures (H2). Additionally, according to previous results (e.g., Dean et al., 2009; Ferreira et al., 2022), it is expected that adjustments will be adequate in terms of specificity and sensitivity for the RDS, TOMM, and CIH-EV, but not for Rey-15IMT (H3). Finally, the selected cut-off points are expected to be lower than those selected for the population with MCI, due to the greater cognitive deterioration characteristic of dementia (H4; McGuire et al., 2019).

## Materials and methods

### Participants

The initial sample had 81 participants (66 women and 15 men); however, five participants were excluded from the cognitively healthy groups, because of the lower Mini-Mental State Examination (MMSE) and/or Montreal Cognitive Assessment (MoCA) results. A final sample of 76 participants (64 women and 12 men) ages 65–94 (*M* = 75.78; *SD* = 0.92) was assessed. The study’s methodology followed an analog simulation design that has been previously used to examine the psychometric properties of validity tests (Simões et al., 2010). As such, participants were either randomly assigned to the control group or feigning group. The control group (*n* = 30, 25 female) was asked to perform to the best of their abilities while the feigning group (*n* = 29, 25 female) was asked to perform as if they had memory impairment due to dementia in order to obtain benefits (such as pension, allowance, or early retirement). The control group presented an age range between 65 and 94 (*M* = 75.20; *SD* = 8.90) and had an average of 8.43 years of education (*SD* = 3.94). The feigning group’s age ranged from 65 to 87 (*M* = 73.31; *SD* = 6.40), and had a mean education of 9.07 years (*SD* = 3.36). In addition to these two groups, a clinical group with a dementia diagnosis (*n* = 17, 14 female) was included in order to improve the generalizability of findings (Rogers, 2008). This diagnosis was previously determined by the medical and neuropsychology team (i.e., a neurologist, a neuropsychologist, and a nurse) at both Residential Facilities. The clinical group presented an age range between 68 and 92 years (*M* = 81.00; *SD* = 6.82) and had an average of 8.35 years of education (*SD* = 3.66).

All participants were required to be age 65 or older, and to be proficient in Portuguese. Participants were excluded if they reported chronic medical issues or illnesses that could compromise cognitive functioning (e.g., convulsions and cerebrovascular disease), traumatic brain injury or substance use, uncorrected visual and/or auditory deficiencies, and/or difficulties with oral comprehension. In addition to these criteria, participants in the control and feigning groups were excluded if they presented with cognitive impairment as demonstrated by MMSE/MoCA results (this was an inclusion criteria of the clinical group who had been diagnosed with dementia). None of the participants in all three groups was involved in judiciary or forensic proceedings.

### Instruments

The following questionnaires and neuropsychological tests were administered: a sociodemographic questionnaire, two screening tests for cognitive impairment (MMSE and MoCA), one embedded performance validity test WAIS-III’s Digit Span Subtest and three stand-alone performance validity tests (TOMM, REY-15IMT, and CIH-EV).

#### Sociodemographic questionnaire

The sociodemographic questionnaire gathered information about the participants’ age, gender, civil status, number of children, residency, economic yield for the family, level of educational, and profession.

#### Mini-Mental State Examination

The MMSE (Folstein et al., 1975; Guerreiro et al., 1994) is a brief test designed to detect global cognitive functioning, specifically in temporal orientation, repetition, attention and calculation, memory, language, and constructive ability. Maximum scores of 30 or higher reflect intact cognitive functioning. For the Portuguese population, established cut-off scores for cognitive impairment are ≤22 for individuals with between 1 and 11 years of schooling, and ≤27 for individuals with over 11 years of schooling (Guerreiro et al., 1994). For dementia patients, however, a cut-off of ≤26 is suggested (e.g., Freitas et al., 2013). For the current study, the MMSE was selected as it is the most commonly used and cited validated cognitive screening test (Simões et al., 2015).

#### Montreal Cognitive Assessment

The MoCA (Nasreddine et al., 2005; Freitas et al., 2011) is a brief screening test for cognitive functioning, specifically executive functioning, visuospatial ability, short-term memory, working memory, attention and concentration, language, and spatiotemporal orientation. It has the capacity to examine milder forms of cognitive deterioration and can discriminate between normative and pathological changes in neuropsychological performance. In agreement with established Portuguese normative scores (Freitas et al., 2011), we applied cut-off scores for cognitive deterioration below 1.5 *SD* for participants 65 years or older, depending on their level of education. As such, cutoff scores for cognitive decline were <16 for individuals with 1–4 years of schooling; <20 for between 5 and 9 years of schooling; <22 for between 10 and 12 years of schooling; and <24 for over 12 years of schooling. Participants in the clinical group with scores lower than 17 were considered to have cognitive decline (Freitas et al., 2013). This measure was selected because, compared to MMSE, it additionally examines executive functioning and is more sensitive to the early signs of dementia (Nasreddine et al., 2005; Freitas et al., 2011).

#### WAIS-III’s Digit Span Subtest

The Digit Span Subtest of the WAIS-III (Wechsler, 1997, 2008) assesses the cognitive domains of memory, attention, and concentration. Evaluators read participants a series of number sequences, which participants must in turn repeat. These numeric sequences progressively increase in length. The Digit Span Subtest includes two conditions: (1) forward repetition, in which participants are asked to repeat the number sequences in the same order in which they were given (cut-off score ≤ 5), and (2) backward repetition, in which participants are asked to repeat the series in the inverse order (cut-off score ≤ 2). The RDS, a calculation proposed by Greiffenstein et al. (1994), is derived from the Digit Span of the WAIS and sums the longest series of digits repeated without committing any errors over the course of two trials (both in the forward and backward repetitions). The RDS cut-off score for the Portuguese population is ≤6 for cognitively normal older adults and ≤5 for MCI (Pinho, 2012). The RDS was selected for this study due to its adequacy in detecting the simulation of neurocognitive deficits (Jasinski et al., 2011).

#### Rey 15-item Memory Test

The Rey-15IMT (Rey, 1964; Boone et al., 2002; Simões et al., 2010) is a PVT that assesses effort and/or the simulation of memory deficits. For the duration of 10 s, participants are presented with a card which includes 15 items of different letters, geometric shapes, and numbers that are displayed across three columns and five rows. In the first trial (Free Recall), participants are asked to draw the items from memory without viewing the card. One point is given for each item participants draw correctly, regardless of where it is drawn on the sheet. In the second trial (Recognition), participants are presented with a card showing 15 of the original items as well as 15 novel items, and are asked to select the original items. Scoring includes the total number of correctly recognized items, the number of false positives (“recognizing” an item when it is novel), and a combined score for the number of correctly recalled items (plus the total of correctly recognized items minus the total false-positives). For the Portuguese population, a cut-off score of <20 has been established for the Combined Result score, and <9 for the Free Recall Trial (Simões et al., 2010). This test was selected as it is a commonly used PVT and has been validated for older Portuguese adults (Simões et al., 2010). Further, convergent validity for the Rey-15IMT has been assessed with other PVTs in Portugal, such as the CIH-EV (Daugherty et al., 2019).

#### Test of Memory Malingering

The TOMM (Tombaugh, 1996; Fernandes, 2009) is a visual forced-choice PVT and is made up of two learning trials in which participants view a series of 50 images of quotidian or common stimuli. Each stimulus is presented for a total of 3 s with 1 s intervals. After the first and second trial, participants are shown the same images from the learning trial intermixed with novel images (distractors). Participants are asked to respond “yes” or “no” as to whether the image was presented in one of the learning trials (hence the forced-choice paradigm). The order of images shown in Trial 1 and Trial 2 are different, and the distractor images vary between both trials, such that the distractor images do not repeat themselves. Feedback is given for each response, and correct responses are given 1 point (resulting in a maximum score of 50 points). The Portuguese cut-off of <45 was used in this study (Fernandes, 2009). The TOMM was selected as it is considered one of the gold-standard PVTs and has been validated for older adults in Portugal (e.g., Fernandes, 2009).

#### Coin in Hand— Extended version

The Coin in Hand test, a forced-choice PVT, was originally designed to assess for simulation of neurocognitive disorders, specifically those presenting with memory complaints (Kapur, 1994). Participants are told that the objective of the test is to examine how memory is influenced by distracting stimuli. Examinees are presented with a pair of opened hands, one of which is holding a coin. Next, the hands close into fists so that the coin is no longer visible, and the participant must count down from 10 before selecting the hand in which he/she saw the coin. Feedback is provided for each response. In response to research suggesting that PVT accuracy improves when multiple levels of perceived difficulty are included (e.g., Bickart et al., 1991; Chiu and Lee, 2002), a new and computerized version of the CIH was developed to include three levels of difficulty (CIH-EV; Daugherty et al., 2019). Each of the three levels of difficulty in the CIH-EV includes 10 trials (30 trials total) in which the coin is randomly presented five times in the left hand and five times in the right hand. To increase the perceived difficulty between trials, the duration of the countdown increases from 10 s in the first trial (“low difficulty”), to 15 in the second (“medium difficulty”), and 30 in the third (“high difficulty”). Before beginning the first trial, participants are cautioned about the different levels of difficulty as the test progresses. The Portuguese cut-offs for the three levels were the same (≤8) for cognitively normal older adults (Ferreira et al., 2022) and young adults (Daugherty et al., 2019). The suggested cut-off point for the total number of hits was ≤26 for cognitively normal older adults (Ferreira et al., 2022) and ≤27 for young adults (Daugherty et al., 2019). The CIH-EV was selected as it is has shown adequate convergent validity, sensitivity, and specificity in the Portuguese population (Daugherty et al., 2019; Ferreira et al., 2022).

### Procedure

Participants in the control and feigning groups were tested at three Senior Universities. After contacting the Universities for permission, we gave a brief presentation about the study’s objectives. Those who were interested signed up to participate. We then randomly assigned participants into experimental groups (control vs. analog) and applied the assessment protocol. Neuropsychological assessment of the clinical group took place at two Residential Care Facilities. All five institutions where data were gathered are located in Lisbon, Portugal. All participants signed an informed consent about the purpose of the study and the type and duration of tasks. After giving consent, each participant was assigned an alphanumeric code to ensure the anonymity of the data.

The neuropsychological protocol, which was individually administered, spanned approximately 50 min in duration for the control group. For the clinical group, the protocol was divided into two sessions of approximately 30 min each. Participants in the control and feigning groups were first screened for exclusion criteria using the Sociodemographic Questionnaire. Inclusion/exclusion information for the clinical group was provided by the Residential Facilities. After completing the cognitive screening using the MMSE and MoCA, participants in the feigning group were instructed to perform the Digit Span subtest, TOMM, REY-15IMT, and CIH-EV while simulating memory impairment typical of dementia in order to obtain pension, allowance, or early retirement. The order of test administration was randomized. The TOMM and CIH-EV were administered using a computer with a 13.3″ screen. In the case of the CIH-EV, all participants were informed about the existence of three difficulty levels, as is suggested in by test authors (Daugherty et al., 2019).

Statistical analyses were performed using IBM SPSS Statistics software.

### Statistical analysis

A Chi-square test was used to compare differences between groups for categorical sociodemographic variables. Independent *t*-tests were used to compare differences between control and feigning groups. They were not used, however, in comparisons with the clinical group because data assumptions were not met due to the reduced sample size of this group. For this reason, a Mann–Whitney U non-parametric test was employed when comparing the feigning and clinical groups.

To accomplish our first aim, a Pearson’s correlation coefficient was used to analyze the relationship between sociodemographic variables, cognitive performance, and the PVTs (RDS, REY-15IMT, TOMM, and CIH-EV) for each group.

Concerning the second and third aim, the adequacy of the cut-off points for the RDS, TOMM, Rey-15IMT, and CIH-EV in discriminating between the feigning and clinical group was analyzed using a receiver operating characteristic (ROC) curve analysis. Specifically, we considered the diagnostic values of specificity and sensitivity and the AUC.

## Results

### Sample characterization

Table 1 presents the sample characteristics for the three groups.

**Table 1:** Sample characteristics for the Control, Feigning, and Clinical groups.

			Control (*n =* 30)	Feigning (*n =* 29)	Clinical (*n =* 17)
Age	m (*SD*)		75.20 (*8.9*)	73.31 (*6.40*)	81 (*6.82*)
Education			8.43 (*3.94*)	9.07 (*3.36*)	8.35 (*3.66*)
Children			1.73 (*1.14*)	1.42 (*0.85*)	1.60 (*1.35*)
Profession		Longest	1.80 (*0.76*)	1.62 (*0.68*)	1.47 (*1.71*)
		Last	1.77 (*0.73*)	1.66 (*0.67*)	1.47 (*0.72*)
Cog Function		MMSE	29.07 (*0*.*17*)	28.86 (*0.18*)	19.9 (*1.36*)
		MoCA	24.67 (*0.49*)	23.69 (*0.41*)	11.41 (*1.15*)
Family yield		≤1,200	*n* (60)	*n* (67.74)	*--*
		1,200–1800	*n* (20)	*n* (29.03)	*--*
		≥1800	*n* (20)	*n* (3.23)	*--*
Sex (female)	*n* (%)		*25* (83.33)	*25* (86.21)	*14* (82.35)
Marital		Married	*13* (43.33)	*11* (37.93)	*3* (17.65)
		Divorced	*9* (30)	*5* (17.24)	*2* (11.76)
		Widowed	*5* (16.6)	*10* (34.48)	*8* (47.06)
		Single	*3* (10.07)	*3* (9.69)	*4* (23.53)
Cohabitation		Alone	*14* (46.67)	*17* (58.06)	*0* (0)
		Partner	*12* (40)	*7* (25.81)	*0* (0)
		Relatives	*3* (10)	*5* (16.13)	*0* (0)
		Residential	*1* (3.33)	*0* (0)	*17* (100)

Children: number of children; Cog Function: Cognitive function as measured by the MMSE and MoCA; Education: years of education; Family yield: economic family yield per month in euros; Profession: the longest and last profession the participants held with a medium level of intellectual stimulation; Residential: living in a Residential facility.

### Analysis of between-group differences in sociodemographic variables and cognitive performance

The Chi-square test compared differences between groups for categorical sociodemographic variables. No significant differences between the three groups were found. *T*-tests were used to compare differences between control and feigning groups for age, MMSE, and MoCA results. No significant differences were found. The Mann–Whitney U non-parametric test was used for the feigning and clinical groups comparison. Significant differences were found for age (*U* = 103.50, *p* < 0.001), and for cognitive function (MMSE: *U* = 37.00, *p* < 0.001; MoCA: *U* = 3.00, *p* < 0.001). As expected, the participants in the clinical group were older and performed worse on the cognitive screening tests.

### Analysis of the relationship between sociodemographic variables, cognitive performance, and PVT performance

The relationship between sociodemographic variables, cognitive performance, and the RDS, REY-15IMT, TOMM, and CIH-EV for each group are presented in Table 2.

**Table 2:** Correlation between sociodemographic variables, neurocognitive functioning, and tests performance for each group.

	RDS	Rey FR	Rey CR	TOMM1	TOMM2	CIH-EV
*Control*						
Sex	0.170	−0.066	−0.028	−0.277	−0.376^*^	−0.09
Age	−0.356	−0.522^*^ ^*^	−0.433^*^	−0.327	−0.159	−0.01
Education	0.645^*^ ^*^	0.307	**0.382** ^*^	−0.157	0.083	0.07
Profession	0.482^*^ ^*^	−0.132	−0.164	0.092	−0.019	−0.29
Last Profession	−0.521^*^ ^*^	−0.068	−0.130	0.112	0.059	−0.29
MMSE	0.326	**0.366** ^*^	0.263	0.205	0.267	0.256
MoCA	0.530^*^ ^*^	**0.456** ^*^	**0.446** ^*^	0.035	0.160	0.109
*Feigning*						
Sex	−0.172	0.000	**0.397** ^*^	0.118	0.056	0.24
Age	0.011	−0.025	0.222	**0.373** ^*^	0.311	0.32
Education	−0.096	**0.390** ^*^	0.090	−0.028	−0.168	−0.07
Profession	−0.093	−0.496^*^ ^*^	−0.039	−0.271	−0.250	0.03
Last Profession	0.014	−0.453^*^	−0.050	−0.230	−0.209	−0.05
MMSE	0.256	0.084	−0.281	0.003	0.008	0.138
MoCA	0.262	0.345	−0.040	0.068	−0.130	0.214
*Clinical*						
Sex	0.473	0.020	0.149	−0.063	−0.072	0.14
Age	0.367	−0.255	−0.253	−0.029	−0.145	−0.09
Education	0.192	0.224	0.397	−0.085	−0.146	0.01
Profession	0.140	−0.215	−0.289	0.103	0.195	−0.10
Last Profession	0.140	−0.215	−0.289	0.103	0.195	−0.10
MMSE	0.356	**0.773** ^*^	**0.603** ^*^	**0.690** ^*^	**0.602** ^*^	0.190
MoCA	0.452	0.688^*^ ^*^	**0.515** ^*^	0.404	0.294	0.265

Rey FR, Rey-15IMT Free Recall; Rey CR, Rey-15IMT Combined Result; TOMM1, First trial of TOMM; TOMM2, Second trial of TOMM; and CIH-EV, Coin in Hand—Extended Version.

* *p* < 0.05. **p < 0.01. Bold values are those for which statistically significant correlations were found.

There were no significant correlations between the CIH-EV and sociodemographic variables and cognitive performance in any of the three groups. All other measures (RDS, REY-15IMT, and TOMM) were sensitive to one or more sociodemographic and/or cognitive variables (see significant coefficients in Table 2).

### Cut-off adjustment analysis

The cutoff scores for were determined using ROC analyses, which produce a comprehensive assessment of diagnostic values for sensitivity and specificity. Further, ROC analyses generate the Area Under the Curve (AUC), a valuable method for evaluating the diagnostic accuracy of a test (Mandrekar, 2010). An AUC of 0.5 is generally considered a non-discriminatory test (incapable of differentiating between clinical vs. non-clinical individuals), an AUC of 0.7–0.8 to be “acceptable,” and an AUC of 0.9 to be excellent (Hosmer et al., 2013). Following the recommended guidelines outlined by Sugarman and Axelrod (2015), selected cut-off points achieving either 50% sensitivity or 90% were also made. Due to the fact that selecting higher or lower cut-off scores will inversely affect sensitivity and specificity (higher sensitivity results in lower specificity, and vice versa), we have followed guidelines to provide a broad range of cut scores with their respective diagnostic values for both sensitivity and specificity (Heilbronner et al., 2009). Considering the grave implications of false-positives in detecting malingering or a lack of effort, we chose to err on the conservative side by selecting cut-off scores with a higher specificity. Thus, while guidelines suggest a specificity of at least 90% (Sugarman and Axelrod, 2015), we have suggested cutoffs that generate a specificity value well above this point. Further, selected cutoffs must generate a specificity value higher than that of the sensitivity.

#### Adjustment analysis of RDS

**Figure 1:**
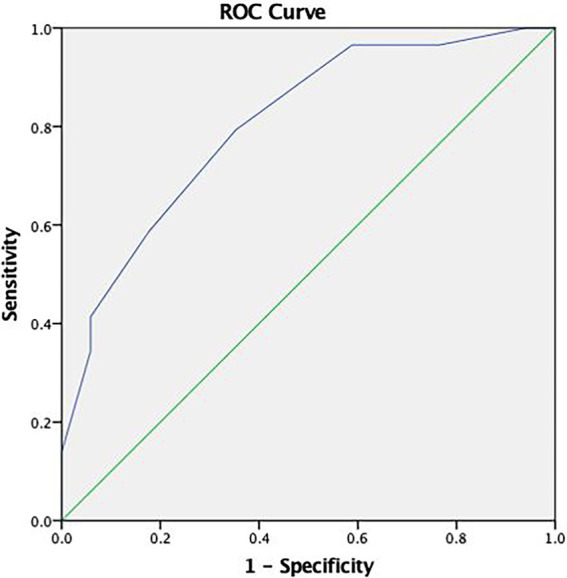
Receiver operating characteristic (ROC) curve concerning the discriminative capacity of the Reliable Digit Span (RDS).

The ROC curve analysis (see Figure 1) revealed a relatively high area under the curve (AUC = 0.804). As such, we selected a cutoff score of either ≤3 or ≤4 for the RDS (see Table 3).

**Table 3:** RDS’ cut-off points sensitivity and specificity for older adults with dementia.

PVT	Cut-off	Sensitivity	Specificity
RDS	≤3	0.41	0.94
	≤4	0.59	0.82
	≤5	0.79	0.65
	≤6	0.97	0.41

PVT, Performance Validity Tests.

Given the considerable difference in sensitivity and specificity between these two cutoffs, we recommend using more than one PVT if the higher cutoff score is to be used (Zenisek et al., 2016).

#### Adjustment analysis of TOMM

**Figure 2:**
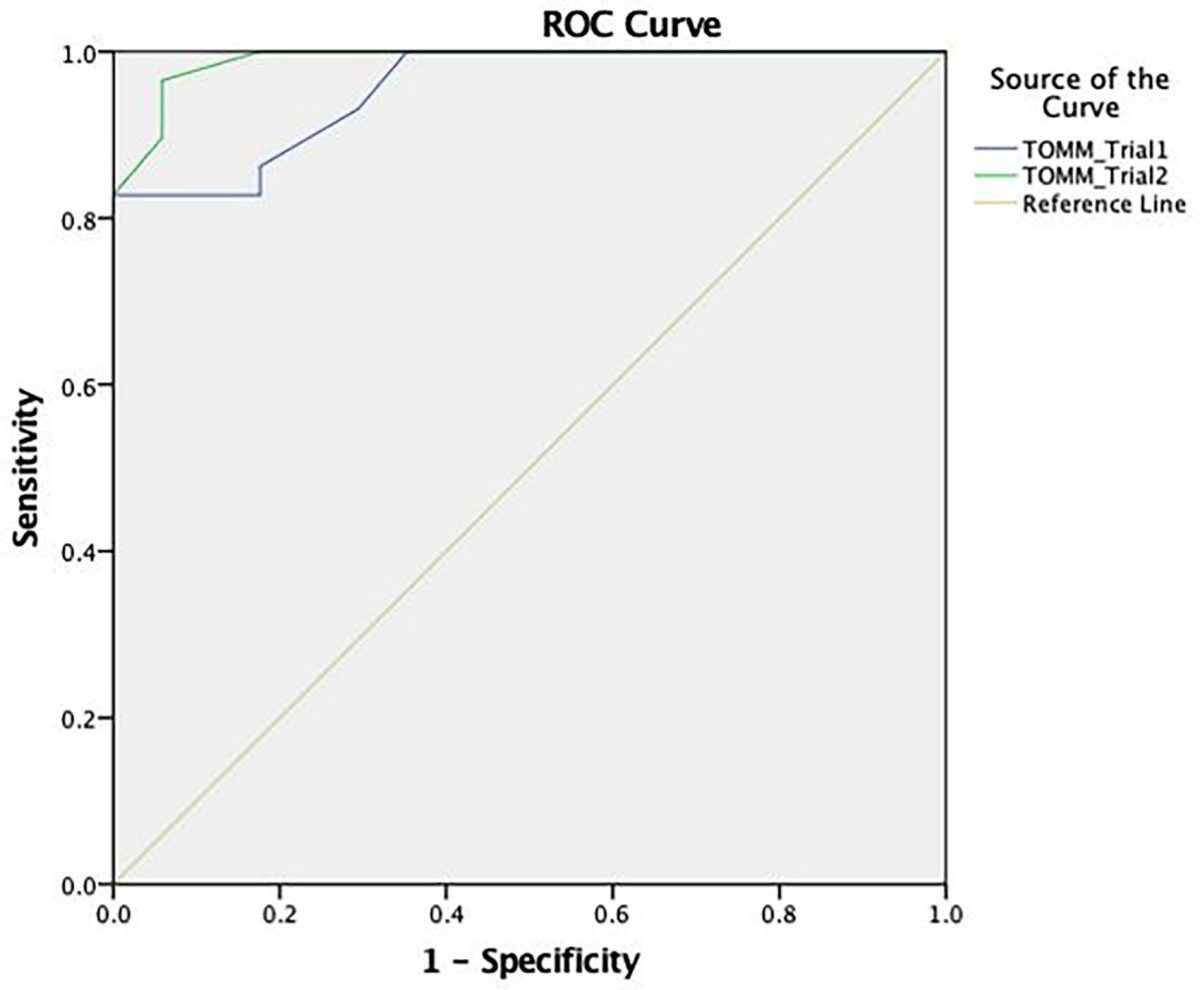
ROC curve concerning the discriminative capacity of the Test of Memory Malingering (TOMM).

Using the ROC curve analysis (see Figure 2), the TOMM demonstrated an adequate capacity in discriminating between the feigning and clinical group with a high area under the curve in Trial 1 (AUC = 0.955) and Trial 2 (AUC = 0.990).

In terms of cut-off scores, the most discriminating score for Trial 1 was ≤26, with a sensitivity of 83% and a specificity of 100%. For Trial 2, the most appropriate cut-off (≤32) indicates a sensitivity of 93% and a specificity of 94% (see Table 4).

**Table 4:** TOMM’s cut-off points sensitivity and specificity for older adults with dementia.

Trial	Cut-off	Sensitivity	Specificity
Trial 1	≤25	0.79	1
	≤26	0.83	1
	≤27	0.83	0.94
Trial 2	≤28	0.83	1
	≤30	0.90	0.94
	≤32	0.93	0.94
	≤34	0.97	0.94

#### Adjustment analysis of REY-15IMT

**Figure 3:**
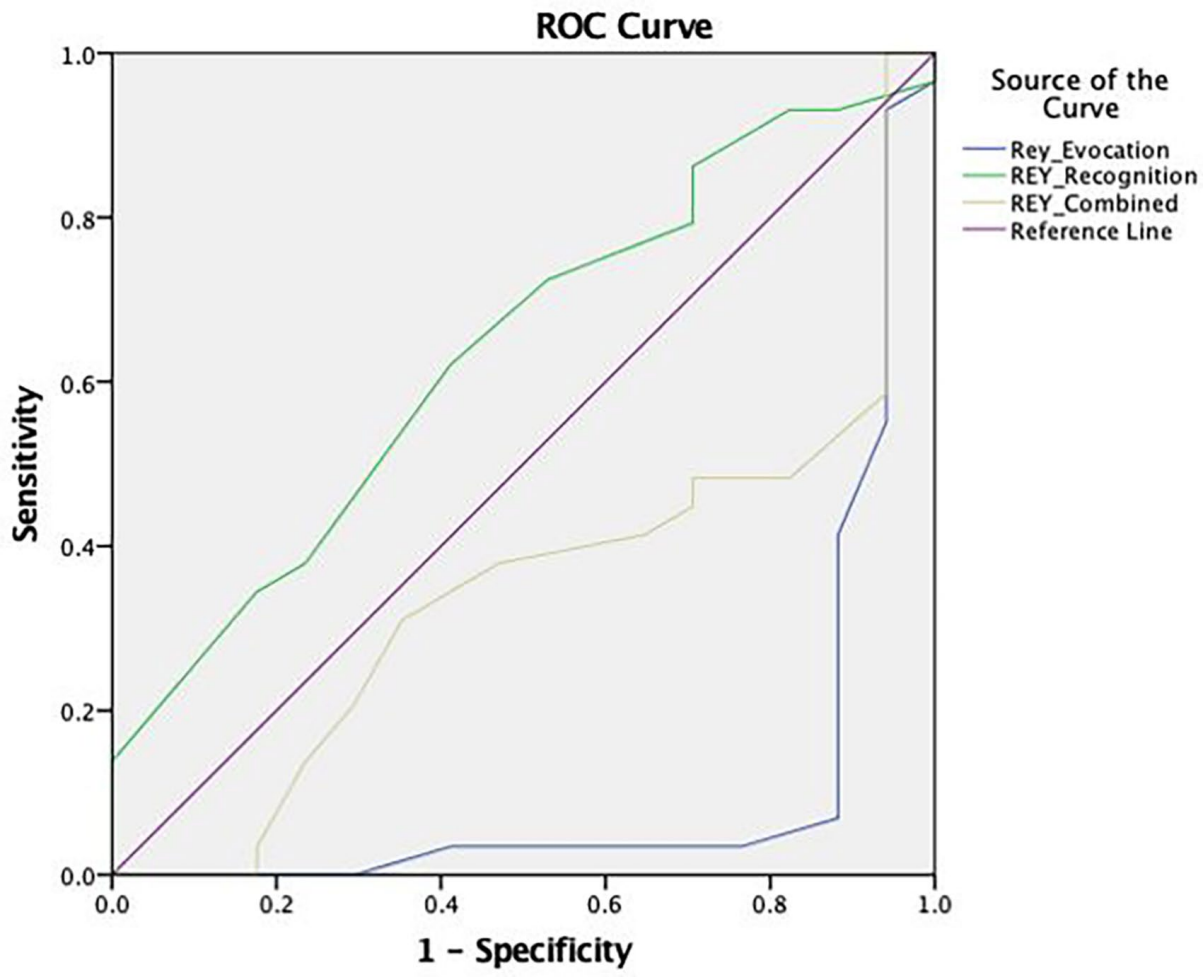
ROC curve concerning the discriminative capacity of the Rey 15-item Memory Test (Rey-15IMT).

The Rey-15IMT (see Figure 3) presented a reduced area under the curve in the Free Recall Trial (AUC = 0.104), the Recognition Trial (AUC = 0.637), and the Combined Result (AUC = 0.345). As this PVT proved to be non-discriminatory, an adjusted cut-off point was not selected.

#### Adjustment analysis of the CIH-EV

For the CIH-EV, a comparison between feigning and clinical groups showed that difficulty levels were discriminative (AUC = 0.952 for level 1; AUC = 0.958 for level 2; and AUC = 0.969 for level 3), thus the most appropriate cut-off points were selected. A total cut-off score of ≤16 for older adults with dementia was selected; with 83% sensitivity and 100% specificity (see Table 5).

**Table 5:** Sensitivity and specificity of CIH-EV cut-off points for older adults with dementia.

Difficulty level	Hits	Sensitivity	Specificity
1	≤3	0.31	1
	≤4	0.69	1
	≤5	0.79	0.94
	≤6	0.93	0.94
	≤7	0.97	0.94
2	≤4	0.38	1
	≤5	0.66	0.94
	≤6	0.97	0.88
3	≤3	0.35	1
	≤4	0.59	0.94
	≤5	0.83	0.94
Total	≤6	1	0.94
	≤14	0.59	1
	≤15	0.76	1
	≤16	0.83	1
	≤17	0.97	0.94

**Figure 4:**
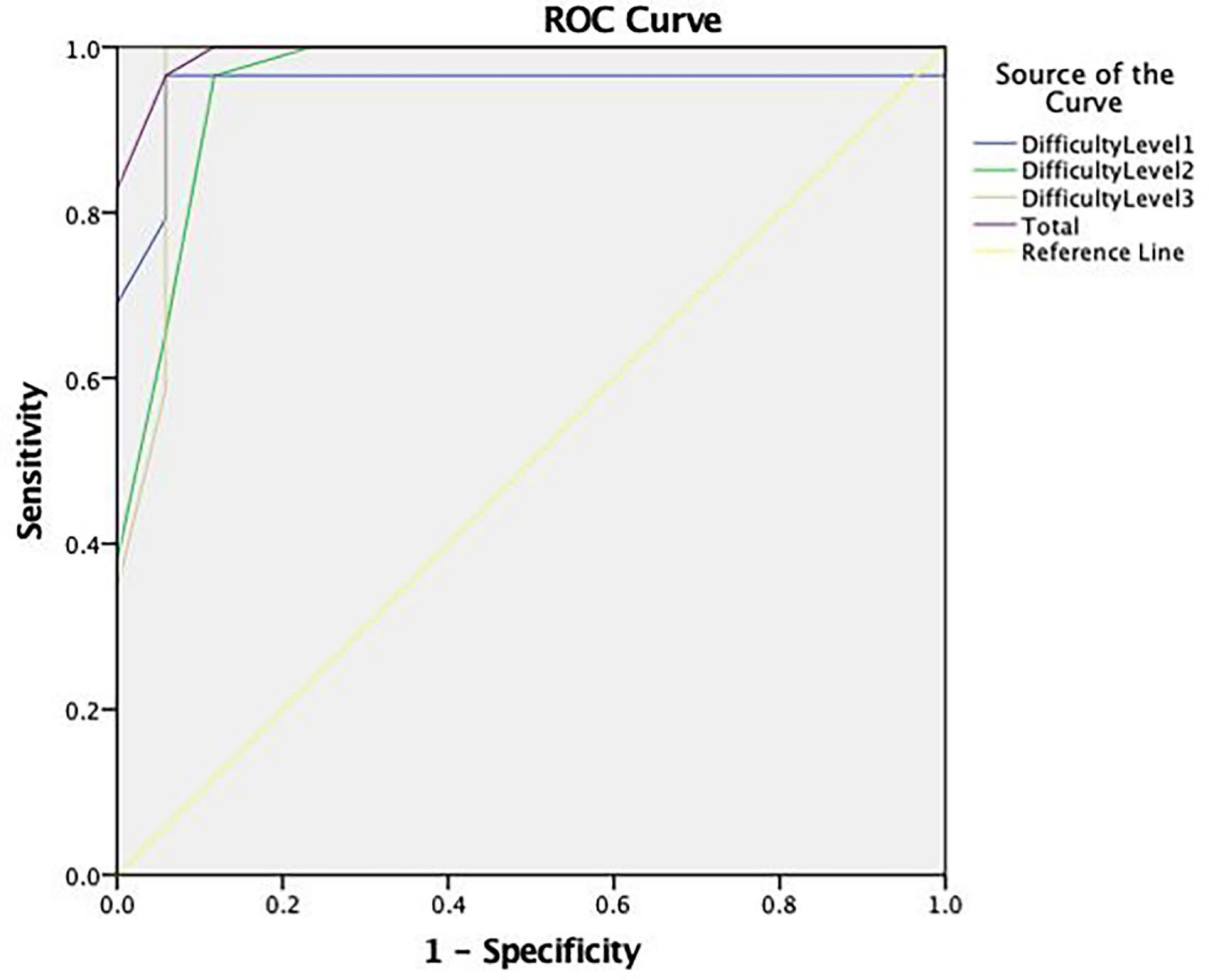
ROC curve concerning the discriminative capacity of the Coin in Hand—Extended Version (CIH-EV).

Regarding performance accuracy (i.e., the total number of hits), the ROC curve analysis (see Figure 4) showed an excellent area under the curve (AUC = 0.993).

### Cut-offs comparison between MCI and dementia population

Descriptive analyses demonstrate that, for Trial 1 of the TOMM, the MCI cut-off of ≤33 (Fernandes, 2009) incorrectly classified 5 (29.4%) clinical dementia patients as performing with insufficient effort, rather than identifying a performance reflective of to cognitive deterioration. No dementia patients, on the other hand, performed below the adjusted cutoff of ≤26 for this clinical population. For Trial 2 of the TOMM, the MCI cutoff of ≤45 (Pinho, 2012) determined 10 dementia participants (58.8%) to perform with insufficient effort, whereas only one dementia patient (0.06%) performed below the adjusted cutoff for dementia of ≤32. Finally, for the RDS, three dementia participants (17.6%) performed with insufficient effort according to the MCI cutoff of ≤5 (Pinho, 2012). When applying the suggested cutoff of ≤6 for dementia patients in Portugal (Pinho, 2012), there were six patients (35.3%) who performed below the cutoff. However, when applying the adjusted cutoffs found in the current study, only one (0.06%) participant performed with insufficient effort with the cutoffs ≤3 and ≤4.

## Discussion

The present study had three principal objectives. First, it aimed to examine the sensitivity of validated PVTs to demographic variables and cognitive functioning in Portugal (RDS, TOMM, REY-15IMT, and CIH-EV). Additionally, it sought to evaluate the need for adjusted cut-off points for dementia patients and, when necessary, provide suggestions for the most appropriate cut-off points for this population according to recommended guidelines (Heilbronner et al., 2009; Sugarman and Axelrod, 2015).

Regarding the first objective and following our first hypothesis (H1), the CIH-EV was the only test to be insensitive to all sociodemographic variables and neurocognitive functioning, both in older adults with dementia and in cognitively normal older adults. This result is similar to those obtained by Schroeder et al. (2012) with the original version of CIH (Kapur, 1994), where performance on the CIH was not associated with neurocognitive functioning, age, education level, nor type of dementia. On the contrary, the remaining PVTs were correlated with at least one sociodemographic variable and/or one neuropsychological test, as expected (H1). Particularly concerning the Rey-15 IMT, in cognitively normal older adults, the Immediate Recall Essay and the Combined Result were associated with age and schooling. Similar results have been reported by Strutt et al. (2011) and Pinho (2012) in cognitively normal older adults. Likewise, the Rey-15IMT was also sensitive to neurocognitive functioning in cognitively normal older adults and the clinical group. In fact, Fazio et al. (2017) had already reported that the Rey-15IMT was sensitive to dementia deficits and states.

Concerning the TOMM, a correlation between Trial 2 and gender was observed in the control group of cognitively normal older adults. A similar result has also been verified by Pinho (2012), albeit for cognitively normal older simulators. Pinho (2012), suggested that women may more prone to insufficient effort, although more research is needed in this area to make this assertion. Regarding age, previous research has reported the influence of age on the TOMM among a group of older adults with dementia (Teichner and Wagner, 2004). While age was not significantly related to TOMM performance in the clinical dementia group in the present study, an association was detected in the group of cognitively normal older adults. The results of the present study, and those previously mentioned, are therefore inconclusive concerning the type of variables that influence TOMM performance. However, they suggest that even “gold standard” tests (Slick et al., 1999; Fazio et al., 2015) may be sensitive to at least one demographic variable in cognitively normal older adults and those with dementia.

Finally, regarding the RDS, performance in the cognitively normal non-feigning group was sensitive to both profession and schooling, the latter of which has also been reported by Choi et al. (2014). Pinho (2012) did not find similar relationships but observed an additional correlation between the RDS and age in a group of cognitively normal older adults. Furthermore, Pinho (2012) observed a relationship between schooling and the RDS, but only for a group of older adults with mild cognitive impairment. The results of the first objective have several implications. First, they provide solid evidence for the CIH-EV (Daugherty et al., 2019) as a valid and sound instrument for effort and simulation detection, considering it was the only instrument included in this study to be insensitive to sociodemographic variables and neurocognitive functioning, both in cognitively normal older adults and in older adults with dementia. Second, the influence of sociodemographic variables on the other PVTs raises concern for the validity and “construct relevance” of these measures, and whether other variables are potentially confounding the measurement [American Educational Research (AERA), American Psychological Association, & National Council on Measurement in Education, 2014].

Concerning our second objective, we examined the discriminating capacity of different cut-off points to evaluate the need for score adjustments for dementia patients in Portugal. As expected (H2; McGuire et al., 2019), it was necessary to adjust cut-off points for dementia patients for the CIH-EV, TOMM, and RDS. On the other hand, the ROC curve analysis suggested that the Rey-15IMT was non-discriminatory, and for this reason, an adjusted cut-off point was not selected for this PVT.

The need to adjust cut-off scores led us to our third goal, which was to select appropriate cut-off points for older adults diagnosed with dementia. Results corroborated our third Hypothesis (H3), where diagnostic values of sensitivity and specificity for the TOMM, RDS, and CIH-EV improved with score adjustments. As expected (H4), the adjusted cut-off points for the TOMM and RDS were lower than those for MCI patients (Table 6). Score adjustment has important practical implications, potentially leading to fewer errors in the detection of insufficient effort and/or simulation. In fact, when applying dementia-specific cutoffs as opposed to MCI cutoffs for Trial 2 of the TOMM, the rate of false-positives dropped by from 58.8 to.06%.

**Table 6:** Comparison of the RDS, TOMM, REY-15IMT, and CIH-EV cut-offs for MCI and dementia.

	RDS	Rey FR	Rey CR	TOMM1	TOMM2	CIH-EVTot
MCI	≤5^*^	1.90^**^	2^**^	≤33^***^	<45^*^	-
Dementia	≤3/4, ≤6^*^	-	-	≤26	≤32	≤16

Rey FR, Rey-15IMT Free Recall; Rey CR, Rey-15IMT Combined Result; and TOMM1 and TOMM2, First and second trial of TOMM, respectively. CIH-EV1, CIH-EV2, and CIH-EV3, CIH-EV difficulty level number; CIH-EVTot, CIH-EV total.

* Pinho, 2012;

** Simões et al., 2010;

*** Fernandes, 2009.

In terms of the RDS, the selected cut-off (≤3 or ≤4) varied greatly from that which has been recommended in previous research for Portuguese dementia patients (≤6; Pinho, 2012). When applying these adjusted cutoffs, false-positive rates dropped from 35.3 to 0.06%. Given that RDS performance was associated with one of the cognitive measures (MoCA) in our study, it is not surprising that a lower cutoff point was needed. While a cut-off of ≤4 would offer greater sensitivity, a cutoff of ≤3 may be preferable in order to err on the side of caution and safe-guard again false-positives. Previous research suggests that using higher cut-offs for the RDS with individuals who have a potential dementia diagnosis increases the likelihood of misinterpreting genuine cognitive impairment as invalid performance (Zenisek et al., 2016). Thus, lower cut-offs may be useful, but only when used in conjunction with other PVTs (Zenisek et al., 2016). In sum, these findings suggest that, when using traditional cut-offs for other diagnostic groups such as MCI patients, high rates of false-positive error may occur. Thus, adjusted cut-offs are needed for dementia patients in order to avoid an incorrect classification of insufficient effort or simulation.

The present study has some limitations. First, the clinical sample is relatively small, preventing us from establishing groups with different levels of dementia severity. Thus, the determined cut-off points may not be adequate for the different levels of deterioration, as lower MMSE scores are associated with increased effort test failure (Dean et al., 2009). Second, the clinical sample is comprised of mostly women, limiting the generalizability of findings. Third, the present study did not include a group of individuals under real suspicion for simulation, with external incentives or secondary gains. Nonetheless, we did include an analog group that was explicitly instructed to feign. While some authors (e.g., Rogers, 2008) consider that the generalization of results from these groups is limited, as they do not have a secondary gain that motivates them, other authors (e.g., Elhai et al., 2007) report no differences between feigning groups with different levels of financial incentives. In light of these limitations, future studies should examine the diagnostic accuracy of PVTs depending on the stage of dementia, as there is evidence that people in mild, moderate, and severe stages obtain different results on PVTs (e.g., Rudman et al., 2011). Further, the use of a feigning group with external incentives or secondary gains may also be useful in determining the discriminating capacity of these measures.

## Conclusion

Our results demonstrated that the CIH-EV was the only PVT included in this study to be insensitive to sociodemographic variables and neurocognitive functioning. The other PVTs (the RDS, Rey 15-Item Test, and the TOMM) were associated with at least one sociodemographic or cognitive variable. Further, the CIH-EV was the least error-prone test for older adults with dementia, providing evidence for its use with this clinical population (Rudman et al., 2011). While more research is needed on how the CIH-EV operates with different levels of cognitive deterioration and in a forensic sample, these results demonstrate that the CIH-EV is a promising instrument with the excellent diagnostic accuracy and discriminatory capacity. In terms of cut-off scores, adjustments were made for dementia patients for the TOMM, CIH-EV, and RDS to improve specificity and sensitivity. No adjustment, however, was made for the Rey-15IMT, which was non-discriminatory between groups. The current findings also suggest that more conservative cutoffs are required in patients with dementia. A diagnostic comparison between MCI cutoffs and adjusted cutoffs for dementia revealed that applying non-representative cutoffs can result in high rates of false-positives. As such, caution should be taken when using cut-off scores that are not specific to the clinical diagnosis.

## Data availability statement

The raw data supporting the conclusions of this article will be made available by the authors, without undue reservation.

## Ethics statement

The studies involving human participants were reviewed and approved by Comissão de Ética e Deontologia, Faculdade de Psicologia, Universidade de Lisboa. The patients/participants provided their written informed consent to participate in this study.

## Author contributions

SF and IF contributed to conception, conceptualization, methodology, analysis and interpretation of data, writing—original draft, and editing. LQ contributed to methodology, formal analysis, interpretation of data, writing—review, and editing. JD contributed to analysis, critically revising, and editing. All authors contributed to the article and approved the submitted version.

## Funding

This work received national funding from FCT—Fundação para a Ciência e a Tecnologia, I. P., through the Research Center for Psychological Science of the Faculty of Psychology, University of Lisbon (UIDB/04527/2020 and UIDP/04527/2020).

## Conflict of interest

The authors declare that the research was conducted in the absence of any commercial or financial relationships that could be construed as a potential conflict of interest.

## Publisher’s note

All claims expressed in this article are solely those of the authors and do not necessarily represent those of their affiliated organizations, or those of the publisher, the editors and the reviewers. Any product that may be evaluated in this article, or claim that may be made by its manufacturer, is not guaranteed or endorsed by the publisher.
